# Supplementary material for: Cloning and Functional Analysis of a Zeaxanthin Epoxidase Gene in Ulva prolifera
Source: Biology (Basel). 2024 Sep 5;13(9):695. doi: 10.3390/biology13090695 (PMC11429058; doi:10.3390/biology13090695)
Supplement: Supplementary file 1 [file biology-13-00695-s001.zip › biology-3173490-supplementary.pdf]

Table S1 Mass spectrometry analysis parameters

Substance Name	Polarity	Parent Ion （m/z）	Product Ion （m/z）	declustering potential (V)	collision energy (V)
Lutein	+	551.5	175.1*/134.9	75	20/25
Zeaxanthin	+	569.5	477.2/175.1	50	18/23
Violaxanthin	+	585.5	567.5/150.9	60	10//55
Antheraxanthin	+	601.3	221.4/583.5	35	30/26

Table S2 Primers and sequences used in this experiment

Gene		Sequence(5 ~ 3)	Purpose
UpZEP	Forward	ATGCAATGGTATGCTTTCCATGAAG	PCR
	Reverse	CTATACTGACACTTTGACCCCA	
pChlamy-*UpZEP*	Forward	GAGATTCGAGGTACCAT ATGCAATGGTATGCTTTCCATGAAGAGA	PCR
	Reverse	CAGAGATCTCTAGAGTGGTGGTGGTGGTG GTGGAAGAGGAAAGGACATACCTCTATGGG	
pYES2-*UpZEP*	Reverse	AGTGTGGTGGAATTC TATGCAATGGTATGCTTTCCATGAAGAGA	PCR
	Reverse	GCCCTCTAGACTCGAGCGTT AGAAGAGGAAAGGACATACCTCTATGGG	
qUpZEP	Forward	GGGGTCCGACATGGACAAAA	qRT-PCR
	Reverse	TGTTGAATGATGCGGTGGGT	
Tubulin	Forward	CTCGCTTCGCTTTGACGGTG	Internal control
	Reverse	CGTGGTACGCCTTCTCGGC	
18SrRNA	Forward	ACCACATCCAAGGAAGGCAGCAG	Internal control
	Reverse	TGCGTCCCACCCGAAATCCAAC	

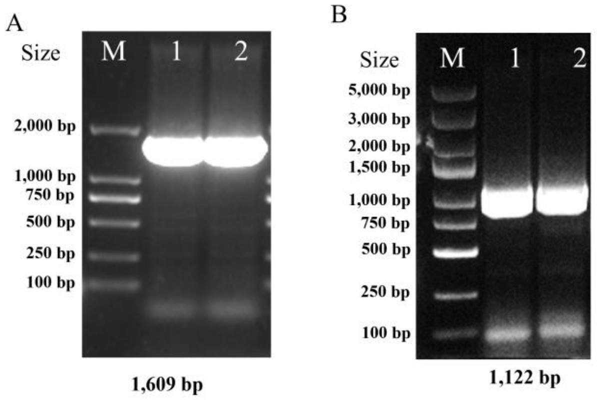

Figure S1. Gel electrophoresis of the cloned product of the *UpZEP* gene. (A) PCR products of full-length sequence of *UpZEP* gene; (B) PCR products of CDS of *UpZEP* gene.
